# Otolaryngologists adhere to evidence-based guidelines for chronic rhinosinusitis

**DOI:** 10.1007/s00405-019-05289-9

**Published:** 2019-01-25

**Authors:** N. M. Kaper, M. C. J. Aarts, P. P. G. van Benthem, G. J. M. G. van der Heijden

**Affiliations:** 1 Department of Otolaryngology, Head and Neck Surgery, University Medical Center Utrecht, Utrecht, The Netherlands; 2 Department of Otolaryngology, Jeroen Bosch Hospital, s’ Hertogenbosch, The Netherlands; 3 Department of Otolaryngology, Head and Neck Surgery, LUMC, University of Leiden, Leiden, The Netherlands; 4 Department of Social Dentistry, Academic Center for Dentistry Amsterdam, University of Amsterdam and VU University, Amsterdam, The Netherlands

**Keywords:** Sinusitis, Practice guideline as topic, Practice guideline, Otolaryngology, Evidence-based practice, Evidence-based medicine, Clinical decision making

## Abstract

**Purpose:**

To assess awareness of, opinion about and adherence to evidence-based guidelines on chronic rhinosinusitis among Dutch Otolaryngologists.

**Methods:**

We assessed implementation of two guidelines, one Dutch and one European, that are both intended for diagnosis and treatment of patients with chronic rhinosinusitis. We invited 485 Otolaryngologists to fill out a questionnaire and report on their opinion on and adherence to the guidelines. The adherence was further tested by 4 clinical case scenarios, derived from guideline recommendations.

**Results:**

166 (34%) completed the questionnaire. 99% of the respondents was aware of one or both guidelines. Most respondents (90%) consider the guidelines as directing or supportive for their clinical practice based on the clinical case scenarios, between 62 and 99% of the respondents act according to guidelines. Concerning diagnosis, CT-imaging is performed more and allergy testing less than recommended. Where multiple treatment options are recommended, the responses are more heterogeneous as a result of this. Nonetheless, high recommended treatment was chosen more often. Otolaryngologists were reluctant in surgical treatment as a first option, which is according to the guidelines.

**Conclusions:**

Overall, both the EPOS and CBO guideline are well known among Dutch Otolaryngologists and 90% indicates that the guideline is important in their daily practice. Adherence to the guidelines is sufficient to high. If multiple treatment or diagnostic options are recommended this leads to a more heterogeneous response pattern. Recommendations with a high grade of recommendation were followed up most often.

**Electronic supplementary material:**

The online version of this article (10.1007/s00405-019-05289-9) contains supplementary material, which is available to authorized users.

## Introduction

Otolaryngologists are both encouraged and expected to incorporate available evidence-based clinical guidelines in daily practice [1]. Their use has become standard of care in most hospitals. For many conditions in Otolaryngology, (inter)national guidelines have been developed [2, 3]. However, the publication of a clinical practice guideline does not immediately result in implementation of the use of the guideline in daily practice [4]. In 1996, a Pathman et al. developed a model for different steps in the implementation of guidelines. Clinicians must be aware of the guideline, agree, adopt the guidelines in clinical practice for their patients, and then actually adhere to the guideline at appropriate times. With each step, there is a risk of losing clinicians in the process of implementing a guideline [5]. After implementation, the guideline can be integraded in a shared decision making approach, with integration of patient specific circumstances or values [6]. Numerous barriers and facilitators of guideline implementation have been identified, that consist of patient, physician, environmental, and guideline-related factors [7–11].

The self-reported adherence to evidence-based guidelines among Dutch Otolaryngologists has been previously assessed, in 2010, and is considered rather high [2]. That is, 62% indicated that their daily practice was supported by guidelines, and 32% stated that guidelines guided their clinical practice.

In this article, we will focus only on guidelines concerning chronic rhinosinusitis (CRS), which is a common disease in the practice of Otolaryngologists, with a reported prevalence ranging from 2 to 11% [12, 13]. For this condition, multiple guidelines have been developed [14–17].

In The Netherlands, the Dutch Society of Otolaryngology and Head & Neck Surgery initiates and maintains evidence-based guidelines for Otolaryngologists [18]. They recommend the use of the Dutch guideline CBO [15]. However, the authors suspect from their own experience that the European guideline, EPOS, is also widely used [16]. The guidelines are available for free on the internet since 2010 and 2012.

In our study, we evaluated the self-reported awareness of the CBO [15] and EPOS [16] guidelines among Dutch Otolaryngologists, their opinion on these guidelines, and the implementation of these guidelines based on clinical scenarios.

## Methods

### Compliance with ethical standards

This study does not involve patients. The Otolaryngologists were approached without obligation and filled out the questionnaire anonymously.

### National survey

Between May 2017 and December 2017, we performed a survey among Dutch Otolaryngologists. We send a questionnaire to 485 Otolaryngologists by e-mail and mail, and the request was repeated twice. The questionnaires took respondents 10–15 min.

### General questions

The first part of the questionnaire related to respondent characteristics, i.e., gender, PhD grade, training in evidence-based practice, time registered as Otolaryngologist, area(s) of interest and how often they read publications related to CRS. We further asked them about the awareness and opinion of both guidelines, the self-reported adherence to the guidelines and reasons not to apply the guidelines [7–11] (see Electronic supplementary material; complete questionnaire).

### Clinical case scenarios

The second part of the questionnaire existed of four clinical case scenarios concerning diagnosis and treatment of patients with CRS. We posed questions on clinical decisions that they would make in these cases and compared their answers to the content and recommendations of the two guidelines, with the intention to provide further insight into the adoption of and adherence to the guideline. The clinical case scenarios were designed to be representative for patients encountered in daily practice.

The recommendations, with corresponding grade of recommendation (GoR), for CBO and EPOS were extracted from the guidelines and compared [19]. EPOS only provides recommendations on treatment options, CBO provides recommendations on both diagnosis and treatment. Therefore, we included questions about both treatment and diagnosis in our clinical case scenarios. For the EPOS guideline, advice regarding diagnosis was retrieved from the full-text of the guideline [16].

The clinical case scenarios are based on patients with (cases 1 and 4) and without nasal polyps (NP) (cases 2 and 3), with (cases 3 and 4) or without (cases 1 and 2) prior treatment. There are four questions concerning diagnosis (1.1, 1.2, 1.3, and 2.1) and four concerning therapy (1.4, 2.2, 3.4, and 4.4).

If provided, the GoR (high, medium, moderate, and low) was extracted from the guideline [19]. The corresponding type of research evidence can be found in Appendix 1 (Electronic supplementary material). We incorporated questions that were based on different GoR, since we expect that responses might be divert. For recommendations with a high GoR, we expect that most respondents will adhere to the guideline, and therefore, clinical practice will show probably little variation. For a lower GoR, we expect less adherence and more variation in adherence to the guideline and in clinical practice. We expect a similar outcome for questions based on contradicting GoRs.

See Table 1 for clinical case scenarios and accompanying questions. See complete questionnaire in Electronic supplementary material for all answer options. Data were analyzed using SPSS (version 21) [20].

**Table 1:** Clinical case scenarios

Clinical case 1
Male, 51 years, presents with decreased smell, purulent rhinorrhea and facial pain in the past 4 months (VAS 4, moderate). The general practitioner has not yet started treatment
1.1 Which anamnestic symptom(s) are a prerequisite to confirm the diagnosis rhinosinusitis?^a^
1.2 Which additional question(s) should you ask your patient?^a^
The patient has no other complaints. Nasal endoscopy shows polyps medial to the middle turbinate
1.3 Which additional examination(s) should you perform?^a^
1.4 How would you treat this patient?^a^
Clinical case 2
A 45-year-old female has complaints of nasal obstruction, post nasal drip and facial pressure in the past 4 months. She has mild complaints and the general practitioner has not yet started treatment. At nasal endoscopy, there are no signs of mucosal disease
2.1 Which additional test(s) should you perform?^a^
2.2 How would you treat this patient?^a^
Clinical case 3
45-year-old female, with complaints of nasal obstruction, post nasal drip and facial pressure in the past 4 months. Despite 6 weeks’ course of intra nasal steroids, her complaints persist. Nasal endoscopy shows purulent discharge medial tot the middle turbinate. Computed tomography shows partially clouded ethmoid and maxillary sinus with obstruction of the osteo-meatal complex
3 How would you treat this patient?^a^
Clinical case 4
The 51-year-old patient with nasal polyps from case 1 has underwent functional endoscopic sinus surgery. However, after 6 weeks, his complaints have returned. On nasal endoscopy, polyps and purulent discharge are visible lateral to the middle turbinate
4 How would you treat this patient?^a^

^a^Multiple answers possible

## Results

### General questions: respondent characteristics

Of the 485 contacted Otolaryngologists, 166 (34%) replied. Five respondents indicated that their discipline was only head and neck surgery. In total, 161 (33.2%) completed the questionnaire, their baseline characteristics did not differ and can be found in Table 2.

**Table 2:** Characteristics of respondents compared to all invited Dutch Otolaryngologists

	Respondents (161) *n* (%)	All Otolaryngologists^a^ (485) *n* (%)
Gender; male	107 (66)	334 (69)
Registry time (years)		
< 10	65 (40)	201 (41)
10–20	53 (33)	156 (32)
20–30	31 (19)	105 (22)
> 30	12 (7)	23 (5)
PhD grade	77 (48)^b^	247 (51)

*n* number, *%* percentage

^a^As provided by the Dutch Society of Otolaryngology and Head & Neck surgery

^b^1 response is missing

Respondents could indicate their area of interest (multiple options possible). The results are described in Table 3.

**Table 3:** Area of interest

Description	*N* (%)
Rhinology	85 (53)
Facial plastic surgery	33 (20)
Otology	86 (53)
OSA/snoring	48 (30)
Pediatrics	45 (28)
Laryngology	17 (11)
Vestibulogy	5 (3)
Skull base surgery	3 (2)

*n* Number of respondents, *%* percentage, *OSA* obstructive sleep apnea

### General questions: evidence-based practice behavior

138 respondents (86%) have had training in evidence-based practice. 61 respondents (39%) indicate that they read publications on rhinosinusitis once a month or more, the remaining respondents read publications on rhinosinusitis less than once a month.

### General questions: awareness of, opinion on, and self-reported adherence to the guidelines

Of the 161 respondents, 1 respondent (1%) was not aware of any guideline. 154 (96%) were aware of the CBO guideline and 119 (74%) of the EPOS guideline. 111 respondents (69%) were aware of both guidelines. Two respondents (1%) were aware of guidelines beside CBO and EPOS, namely, the guideline by Rosenfeld [14].

Of the 154 Otolaryngologists aware of the CBO guideline, the guideline is used daily by 60 respondents (39%), 2–3 times a week by 36 respondents (23%), and once a week or less by 41 respondents (27%). Of the 119 respondents aware of the EPOS guideline, 35 (29%) uses it every day, 34 (29%) 2–3 times a week, and 45 (39%) uses it once a week or less.

The CBO guideline is directing practice for 37 (24%) respondents, supportive for 100 (65%) and impeding for three (2%). Of the remaining respondents; three (2%) do not know, two (1%) think of the guideline as “an opinion”, and 5 (3%) state that it should be revised. The EPOS guideline is directing practice for 34 respondents (29%), supportive for 72 (61%) and impeding for 5 respondents (4%). Three respondents (3%) state that the guideline should be revised. The opinion of the respondents on the content of the guideline can be found in Table 4. For CBO guideline, 115 (75%) respondents think that it is most or partly based on clearly traceable sources, for EPOS this is similar, 91 respondents (76%).

**Table 4:** Opinion on the content of the guideline

Recommendations are based on clearly traceable sources	CBO *n* (%)	EPOS *n* (%)
Yes	69 (45)	50 (42)
Partly	46 (30)	41 (34)
No	8 (5)	4 (3)
Don’t know	31 (20)	24 (20)

For CBO total amount of respondents is 154, for EPOS 119

*n* number of respondents, *%* percentage

**Fig. 1:**
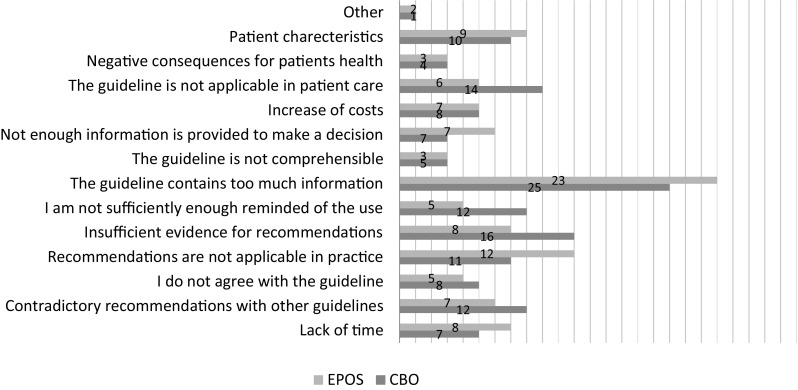
Barriers to guideline application (respondents could choose multiple answers). Other: prefers the other guideline. The numbers indicate the number of respondents, the *y*-axis percentages (1–50%). CBO; total of 93 respondents (60%), EPOS; total of 74 respondents (62%). Created with Microsoft Excel version 16

To provide more insight in the opinion of the respondents on the two guidelines, we asked them to indicate barriers to application of the guideline (despite being aware of it) [7–11] and their answers can be found in Fig. 1. There are no differences between the guidelines. All selected barriers play a role, but the largest barrier experienced by our respondents is the amount of information provided in the guidelines.

### Clinical case scenarios

The clinical case scenarios with accompanying questions can be found in Table 1. The answers to the questions were compared to both guidelines. For question 1.1, EPOS and CBO use the exact same definition (see Electronic supplementary material Appendix 2), and they do not provide a grade of recommendation to this definition. As expected, most respondents (143; 89%) use the correct definition of anamnestic symptoms and there was not much variation in answer options.

For question 1.2, the responses can be found in Table 5. For symptoms that both guidelines advise to check (asthma, allergy, smoking), most respondents comply. The symptoms not mentioned by one or two guidelines (passive smoking and viral infections), are far less frequently asked. Note that reflux is not recommended to ask by both guidelines; nevertheless, one-third of respondents asks this anyway.

**Table 5:** Answers to question 1.2 (multiple answers possible)

Symptoms	GoR	Guideline	*n* (%)
Symptoms related to asthma (+)	NP	EPOS	142 (94)
	Medium	CBO	
Symptoms related to allergy (+)	NP	EPOS	158 (98)
	Moderate	CBO	
Reflux (−)	NP	EPOS	46 (29)
	Moderate	CBO	
Smoking (+)	NP	EPOS	114 (71)
	Moderate	CBO	
Passive smoking (+)	NA	EPOS	45 (30)
	NA	CBO	
Viral airway infections (+)	NP	EPOS	47 (29)
	NA	CBO	

+ Advised to ask, − advised not to ask, *GoR* grade of recommendation, *Medium* one study of A2 or at least two independent studies of B, *Moderate* one study of B or C. (see Electronic supplementary material Appendix 1), *NP* advised in guideline, grade of recommendation not provided; EPOS does not provide recommendations for diagnosis (like CBO), the answers are retrieved from the text and do, therefore, not have a grade of recommendation, *NA* not mentioned in guideline

Question 1.3 (see Table 1) is on diagnostics tests for a patient with complaints of CRS, with polyps seen by nasal endoscopy. The guidelines give multiple recommendations, with varying GoR, and we also see variation in answers between respondents. 73 respondents (45%) would perform no additional tests, corresponding to both guidelines. 46 (29%) would perform a CT scan, even though it is not advised by both guidelines (EPOS; no GoR, CBO; moderate/low). It is advised only pre-operative or when in doubt about diagnosis in patients with persistent complaints. Allergy testing is advised by both guidelines for patients with anamnestic symptoms of allergy (EPOS: no GoR, CBO: moderate), and 66 (41%) of respondents would perform the test for this patient. No respondent would perform an X-ray or a maxillary sinus culture, and this corresponds with the advice of both guidelines (EPOS: no GoR, CBO moderate). 11 respondents (7%) would perform a nasal culture. This is not mentioned by EPOS and not advised by CBO (GoR medium). In total, 114 (71%) of respondents act according to the guideline(s).

Question 1.4 (see Table 1) discusses treatment options for a patient with CRS and nasal polyps. Answers and guideline recommendations can be found in Table 6. Both guidelines advise intranasal steroids with high recommendation, which is followed by 93%. In total, 141 (88%) act of respondents act according to the guideline(s). Striking is that nasal saline irrigation is applied by 79% of respondents, while it is only mentioned by 1 guideline with a low GoR.

**Table 6:** Treatment for patients with CRS with and without nasal polyps

Treatment option	Guideline	1.4 CRS (with nasal polyps)			2.2 CRS (without nasal polyps)		
		Recommendation	GoR	*n* (%)	Recommendation	GoR	*n* (%)
None	EPOS	NA	NA	1 (1)	NA	NA	5 (3)
	CBO	NA	NA		NA	NA	
Nasal saline irrigation	EPOS	+	Low	127 (79)	+	High	123 (76)
	CBO	NA	NA		+	High	
Short course of antibiotics	EPOS	+	Moderate	52 (32)	−^a^	Medium	13 (8)
	CBO	−^a^	Moderate		−^a^	Moderate	
Long-term course of antibiotics	EPOS	+	Moderate	9 (6)	+	Moderate	0
	CBO	−	Moderate		−	Moderate	
Intranasal steroids	EPOS	+	High	150 (93)	+	High	142 (88)
	CBO	+	High		+	High	
Systemic steroids	EPOS	+	High	67 (42)	+	NP	2 (1)
	CBO	+	High		NA	NA	
Decongestants	EPOS	−	Low	10 (6)	−	Low	10 (6)
	CBO	−	Medium		−	Medium	
FESS^b^	EPOS	−	NP	4 (2)	−	NP	1 (1)
	CBO	−	Moderate		−	Moderate	
Antihistamines^c^	EPOS	−	Medium	8 (5)	−	Medium	0
	CBO	−	Low		−	Low	
Other^d^	Polyp extraction			1 (1)	“		0
	Nasal ointment			1 (1)	“		1 (1)
	Await CT result			0	“		5 (3)
	Coblation of inferior turbinate			0	“		1 (1)

+ Treatment is recommended, − treatment is not recommended, “ idem, *GoR* grade of recommendation, *n* number of respondents, *%* percentage of total, *NP* advised in guideline, grade of recommendation not provided, *NA* not mentioned in guideline, *High* research based on A1 or two independent A2 studies, *Medium* one study of A2 or at least two independent studies of B, *Moderate* one study of B or C, *Low* expert opinion (see Electronic supplementary material Appendix 1)

^a^Only recommended for acute exacerbations

^b^FESS is only recommended after failure of conservative treatment by both guideline (both with and without polyps)

^c^Antihistamines are only recommended in patients with positive allergy testing (both with and without polyps)

^d^Not mentioned in CBO nor EPOS

Clinical case 2 concerns a patient with CRS without mucosal disease at nasal endoscopy (see Table 1). For question 2.1, the respondents indicate the diagnostic test(s) that they would perform. The guidelines recommend multiple diagnostic strategies and we see this reflected in the varied response. 68 respondents (42%) would not perform additional testing. 56 (35%) would perform a CT scan. 57 (35%) would perform allergy testing. According to the guidelines, all these choices are justified. The recommendations are identical for question 1.3. One (1%) respondent would perform an X-ray, one (1%) a maxillary sinus culture, one (1%) an X-OPT, and two (1%) a nasal culture. This does not correspond with both EPOS or CBO (see question 1.3 for recommendations). In total, only 3% would perform diagnostic tests that were not advised by the guidelines.

For question 2.2, the answers are displayed in Table 6. In total, 107 (66%) of respondents act according to the guideline(s). Most respondents chose the answer option with a high GoR (intranasal steroids and saline irrigation).

Clinical case 3 concerns a patient with CRS without nasal polyps, confirmed by both nasal endoscopy and CT, that has been treated with intranasal steroids. For guidelines recommendations, see Table 6 (2.2). Note that this patient can be considered as having an exacerbation. There are multiple treatment options according to the guidelines, this is reflected in a varied response. Most respondents chose 3 or more treatment modalities. 60 (37%) would start nasal saline irrigation, 98 (61%) would start short-term antibiotics and 22 (14%) long-term. Concerning long-term antibiotics, the guidelines provide a contradicting recommendation. 99 (62%) would continue intranasal steroids, and 49 (30%) would start systemic steroids. All these treatment options are considered valid according to the guidelines. 24 (15%) would start decongestants (in combination with another treatment option) and 29 respondents (18%) would choose FESS, both treatment options are not according to guideline recommendations. In total, 113 (62%) of respondents act according to the guideline(s).

Clinical case 4 concerns a patient with CRS and nasal polyps that has been operated but still has complaints, with sign of disease at nasal endoscopy. For guidelines recommendations, see Table 6 (1.4). Note that this patient can be considered as having an exacerbation. Multiple treatment options are possible according to the guidelines, and this is reflected by a varied response. Most respondents choose a combination of 3 or more treatment options. 124 (77%) would treat this patient with saline irrigation and 137 (85%) with intranasal steroids). 63 (39%) would start short-term antibiotics, 28 (17%) long-term. 96 (60%) would start systemic steroids. All these treatment options correspond to the guideline(s) (except for long-term antibiotics according to CBO). High recommended treatment was chosen more often, while treatment option with moderate, low, or contradicting recommendations is chosen less often. 2 (1%) opt for revision surgery. Both guidelines state that revision surgery is reserved for patients failing maximal conservative therapy (EPOS: no GoR, CBO: Moderate GoR). In total, 159 (99%) of respondents act according to the guideline(s).

## Discussion

### Synopsis of key findings

Guidelines on rhinosinusitis are very well known by Dutch Otolaryngologists. The guideline recommended by the Dutch Society of Otolaryngology and Head & Neck Surgery, the CBO guideline [15] is best known, but also the EPOS guideline [16] is very well known, as we expected in advance. Most respondents (99%) are aware of one or two guideline and the majority indicates that they have confidence in the guideline. The self-reported adherence to the guideline is high, 60% of respondents uses the guideline every day or 2–3 times a week, and 90% indicates that the guideline is leading or supportive in their daily practice. Concerning the clinical case scenarios, 62–99% respondents responded according to the guidelines, which we consider to be sufficient to good adherence. For diagnosis of CRS with polyps, CT-imaging is performed more and allergy testing less than recommended. It is also noticeable that if multiple or contradicting treatment options are recommended, the overall response is more heterogeneous as a result of this. Nonetheless, high recommended treatment was chosen more often. In general, surgical treatment is not chosen as first option, which is according to both guidelines.

### Limitations of the study

Some limitations can be addressed. First, our response rate was 34%, which means that a selection of Otolaryngologists has responded. This could be a selection that known the topic and guideline(s) very well and is positive about the use, or vice versa. We believe that our sample is representative of all Dutch Otolaryngologists, since their baseline characteristics do not differ (Table 2). When we look at the specific interest of our respondents (Table 3), this shows a varied group, which is also a resemblance of Dutch Otolaryngologists.

Second, we tried to assess adherence to the guideline according to questions based on clinical case scenarios. Overall, 62–99% respondents responded according to the guidelines, which we consider to be sufficient to good adherence. This might not reflect their actual clinical behavior, since there is the possibility of socially desirable answers, and since it was possible for respondents to check the guideline for the right answers while filling out the questionnaire. The actual adherence could be lower than we found in this study. In clinical practice, less participants might act according to the guidelines.

Third, this study focusses on the question if the recommendations of the guidelines are implemented. The actual purpose of the guideline, i.e., whether it has led to better treatment outcomes, has not been studied by us [3]. This is a different research question and could be interesting for further research on this topic.

### Comparison to other studies

In the study of Aarts et al. [2], 61% of Dutch Otolaryngologists reported to have accurate knowledge of the CBO guideline, our results show adherence has improved since then. Other studies on the implementation of CBO or EPOS have not been performed. A study on the implementation of the guideline by Rosenfeld [14] based on retrospective chart review of 10 Otolaryngologists showed that adherence to recommendations on rhinosinusitis ranged from 4 to 88% and concluded that adherence was overall rather low [21]. Our results show better adherence, but it needs to be considered that self-reported adherence and clinical practice might not correspond [3].

### Implications for future guideline development

Based on the results of our survey, we formed recommendations for the future development or revisions of the guidelines. As we expected from the previous literature, we also found multiple and various barriers to the use of the guidelines [7–11]. Of our respondents, 60% indicated reasons not to apply the guidelines (see Fig. 1). For both the CBO and EPOS guidelines, the most important barrier was that they contain too much and too detailed information. Therefore, authors of guidelines have to watch for concise information, clear recommendations, and a manageable guideline.

Because of the regularly expected need for actualization, guidelines have an “expiry date”, after that date, the guideline is no longer valid [22]. This could apply to both guidelines, since they date from 2010 to 2012. Recommendations could be outdated or overtaken by new publications. We, therefore, advise authors to update the guideline regularly, with a maximum of 5 years [22].

In advance, we expected that on topics, where the guideline was very clear and directive, there would be little variation in answers. For topics, where the guidelines give multiple options or are contradictive, we expected more variation in response. With a few exceptions, our results mostly confirmed this. Therefore, we recommend authors to formulate their recommendations in a way that the content and intention are clear to the reader. Authors should also take into account the fact that recommendations with lower GoR might be followed up less than recommendations with high GoR.

We found contradictive recommendations between guidelines, e.g., long-term antibiotics for the treatment of CRS. It is not desirable that there are differences between guidelines, since this is confusing for its reader. Our results show this, because there in case of contradicting recommendations, we found more variation in practice and less adherence. Authors of guidelines should compare their recommendations with those of other guidelines (if available) to check for contradictions.

## Electronic supplementary material

Below is the link to the electronic supplementary material.

Supplementary material 1 (DOCX 25 KB)
