# Supplementary material for: Otolaryngologists adhere to evidence-based guidelines for chronic rhinosinusitis
Source: Eur Arch Otorhinolaryngol. 2019 Jan 25;276(4):1101–8. doi: 10.1007/s00405-019-05289-9 (PMC6426812; doi:10.1007/s00405-019-05289-9)
Supplement: Supplementary file 1 — Supplementary material 1 (DOCX 25 KB) [file 405_2019_5289_MOESM1_ESM.docx]

**Appendix 1. Ratings of two evidence-based guidelines for level of evidence and grade of recommendation into a uniform rating**

| EPOS 2012^16^ | CBO 2010^15^ | Uniform | | | | |
| --- | --- | --- | --- | --- | --- | --- |
| Level of evidence | | | | | | |
| Ia = Evidence from meta-analysis of randomized controlled trials  Ib = Evidence from at least one randomized controlled trial  IIa = Evidence from at least one controlled study without randomization  IIb = Evidence from at least one other type of quasi-experimental study  III = Evidence from non-experimental descriptive studies, such as comparative studies, correlation studies, and case-control studies  IV = Evidence from expert committee reports or opinions or clinical experience of respected authorities, or both | A1 = Systematic review of at least two independent executed research of level A2  A2 = For intervention a randomized controlled trial, double blind, and good quality. For diagnostic, the use of a golden standard and a large enough research population. For prognosis and etiologies, a prospective cohort study with restricted follow-up, controlled for confounding and a large enough research population.  B = For intervention a comparative study, but without the point noted in A2. For diagnoses a comparative study, but not the golden standard. For others a prospective cohort study, but without the points noted in A2.  C = Non-comparative studies.  D = Expert opinion. | Ia | A1 | | Excellent | |
|  |  | Ib | A2 | | High | |
|  |  | IIa, IIb | B | | Medium | |
|  |  | III | C | | Moderate | |
|  |  | IV | D | | Low | |
| Grade of recommendation | | | | | | |
| A = Directly based on category I evidence  B = Directly based on category II evidence or extrapolated recommendation from category I evidence  C = Directly based on category III evidence or extrapolated recommendation from category I or II evidence  D = Directly based on category IV evidence or extrapolated recommendation from category I, II or III evidence | 1 = Research based on A1 or two independent A2 studies.  2 = One study of A2 or at least two independent studies of B.  3 = One study of B or C.  4 = Expert opinion. | A | | 1 | | High |
|  |  | B | | 2 | | Medium |
|  |  | C | | 3 | | Moderate |
|  |  | D | | 4 | | Low |

**Appendix 2. Definition of CRS in CBO and EPOS**

Chronic Rhinosinusitis (with or without NP) in adults is defined as:
Presence of two or more symptoms one of which should be either nasal blockage/obstruction/congestion or nasal discharge (anterior/posterior nasal drip):
± Facial pain/pressure;
± reduction or loss of smell;
for ≥12 weeks;

**Supporting information**

**Complete questionnaire (translated from Dutch)**

**Survey on chronic rhinosinusitis**This survey consists of two parts. The first part is about your background and your opinion on the current guidelines for chronic rhinosinusitis. The second part concerns short clinical case scenarios on chronic rhinosinusitis with corresponding questions about diagnosis and treatment.

**Background information**

**What is your gender?**

○ Male

○ Female

**How long have you been an otolaryngologist?**

○ 0-10 years

○ 10-20 years

○ 20-30 years

○ Longer

**Do you have a PhD?**

○ Yes

○ No

**Were you trained in "evidence-based practice"?**

○ Yes

○ No

**What is your subspecialty/which area is of particular interest to you? (multiple answers possible)**

○ Rhinology

○ Facial plastic surgery

○ Head and neck surgery

○ Otology

○ Obstructive Sleep Apnea and snoring

○ Pediatric ENT

○ Other (please give further explanation);…………………………………………………………………………….

……………………………………………………………………………………………………………………………………………….

**Background information**

**How often do you read recent scientific publications on rhinosinusitis?**

○ Every day

○ 2-3 times a week

○ Once a week

○ Once a month

○ Once every 3 months

○ Less than once every 3 months

**Questions about your use of the guideline(s)**

**Which evidence-based guidelines on chronic rhinosinusitis do you know?**

○ Chronic rhinosinusitis and nasal polyps (CBO 2010, Dutch guideline)

○ European Position Paper on Rhinosinusitis and Nasal Polyps (EPOS 2012)

○ Other (please give further explanation);……………………………………………………………………………..

………………………………………………………………………………………………………………………………………………..

**What are your reasons not to apply the recommendations from the CBO 2010 guideline?** **(multiple answers possible)**

○ I do not use the guideline

○ I don’t have enough time to read the guideline

○ The recommendations conflict with other guidelines

○ I don’t agree with the guideline

○ The recommendations are not applicable in daily practice

○ There is insufficient evidence for the recommendations

○ I am not sufficiently reminded of the use of the guideline

○ The guideline contains too much information

○ The guideline is incomprehensible

○ Too little information is provided to make a decision

○ Working according to the guideline increases patient costs

○ The guideline is not applicable in patient care

○ The recommendations have negative health consequences for patients

○ Other (please give further explanation); …………………………………………..…………………………………

…………………………………………………………………………………………………………………………………………………

**What are your reasons not to apply the recommendations from the EPOS 2012 guideline?** **(multiple answers possible)** ○ I do not use the guideline

○ I don’t have enough time to read the guideline

○ The recommendations conflict with other guidelines

○ I don’t agree with the guideline

○ The recommendations are not applicable in daily practice

○ There is insufficient evidence for the recommendations

○ I am not sufficiently reminded of the use of the guideline

○ The guideline contains too much information

○ The guideline is incomprehensible

○ Too little information is provided to make a decision

○ Working according to the guideline increases patient costs

○ The guideline is not applicable in patient care

○ The recommendations have negative health consequences for patients

○ Other (please give further explanation); …………………………………………..…………………………………

…………………………………………………………………………………………………………………………………………………

**What is your opinion about the recommendations in the guideline CBO 2010?**

○ Contains clearly retrievable sources for most the recommendations

○ Contains clearly retrievable sources for part of the recommendations

○ Contains unclear sources for most the recommendations

○ Other (please give further explanation);……………………………..……………………………………………….

…………………………………………………………………………………………………………………………………………………………….

**What is your opinion about the recommendations in the guideline EPOS 2012?**

○ Contains clearly retrievable sources for most the recommendations

○ Contains clearly retrievable sources for part of the recommendations

○ Contains unclear sources for most the recommendations

○ Other (please give further explanation);……………………………..……………………………………………….

…………………………………………………………………………………………………………………………………………………………….

**What is your opinion about the guideline CBO 2010?**

○ The guideline is directing in my practice

○ The guideline supports my practice

○ The guideline impedes my practice

○ Other (please give further explanation);……………………………..……………………………………………….

…………………………………………………………………………………………………………………………………………………………….

**What is your opinion about the guideline EPOS 2012?**

○ The guideline is directing in my practice

○ The guideline supports my practice

○ The guideline impedes my practice

○ Other (please give further explanation);……………………………..……………………………………………….

…………………………………………………………………………………………………………………………………………………………….

**How often do you use the guideline CBO 2010?**

○ Every day

○ 2-3 times a week

○ Once a week

○ Less than once a week

○ Other (please give further explanation);……………………………..………………………………………………

…………………………………………………………………………………………………………………………………………………………….

**How often do you use the guideline EPOS 2012?**

○ Every day

○ 2-3 times a week

○ Once a week

○ Less than once a week

○ Other (please give further explanation);……………………………..………………………………………………

…………………………………………………………………………………………………………………………………………………………….

**In case you are using a different guideline, how often do you use it?**

○ Every day

○ 2-3 times a week

○ Once a week

○ Less than once a week

Clinical case scenarios

***Clinical case 1***Male, 51 years old, presents with decreased smell, purulent rhinorrhea and facial pain in the past 4 months (VAS 4, moderate). The general practitioner has not yet started treatment.

**1.1 Which anamnestic symptom(s) are a prerequisite to confirm the diagnosis rhinosinusitis? (multiple answers possible)**

○ Nasal obstruction or rhinorrhea (anterior and/or posterior)

○ Rhinorrhea (anterior and/or posterior)

○ Nasal obstruction

○ Facial pain/pressure

○ reduction or loss of smell

○ Coughing

**1.2 Which additional question(s) should you ask your patient, before you proceed to physical examination? (multiple answers possible)**

○ Symptoms resembling asthma

○ Symptoms resembling allergy

○ Symptoms resembling GERD/reflux

○ Smoking

○ Passive smoking

○ The occurrence of viral airway infections

***Clinical case 1, continuation***The patient has no other complaints. Nasal endoscopy shows polyps medial to the middle turbinate.

**1.3 Which additional examination(s) should you perform? (multiple answers possible)**

○ None

○ Computed tomography of the nasal sinuses

○ X-ray of the nasal sinuses

○ Culture from the middle nasal passage

○ Maxillary sinus culture

○ Allergy test

○ Other (please give further explanation);……………………………..………………………………………………

…………………………………………………………………………………………………………………………………………………………….

**1.4 How would you treat this patient? (multiple answers possible)**

○ No treatment required

○ Nasal saline irrigation

○ Short course of antibiotics (<4 weeks)

○ Long term course of antibiotics (>12 weeks)

○ Intranasal corticosteroids

○ Systemic corticosteroids

○ Local decongestants

○ FESS (Functional endoscopic sinus surgery)

○ Antihistamines

○ Other (please give further explanation);……………………………..………………………………………………

…………………………………………………………………………………………………………………………………………………………….

***Clinical case 2***A 45-year-old female has complaints of nasal obstruction, post nasal drip and facial pressure since 4 months. She has mild complaints and the general practitioner has not yet started treatment. At nasal endoscopy, there are no signs of mucosal disease.

**2.1 Which additional test(s) should you perform? (multiple answers possible)**

○ None

○ Computed tomography of the nasal sinuses

○ X-ray of the nasal sinuses

○ Culture from the middle nasal passage

○ Maxillary sinus culture

○ Allergy test

○ Other (please give further explanation);……………………………..………………………………………………. …………………………………………………………………………………………………………………………………………………

**2.2 How would you treat this patient? (multiple answers possible)**

○ No treatment required

○ Nasal saline irrigation

○ Short course of antibiotics (<4 weeks)

○ Long term course of antibiotics (>12 weeks)

○ Intranasal corticosteroids

○ Systemic corticosteroids

○ Local decongestants

○ FESS (Functional endoscopic sinus surgery)

○ Other (please give further explanation);……………………………..…………………………………………………………..

…………………………………………………………………………………………………………………………………………………………….

***Clinical case 3***45-year-old female, complaints of nasal obstruction, post nasal drip and facial pressure in the past 4 months. Despite 6 weeks’ course of intra nasal steroids, her complaints persist. Nasal endoscopy show purulent discharge medial tot the middle turbinate. Computed tomography shows partially clouded ethmoid and maxillary sinus with obstruction of the osteo-meatal complex.

**3.1 How would you treat this patient? (multiple answers possible)**

○ No treatment required

○ Nasal saline irrigation

○ Short course of antibiotics (<4 weeks)

○ Long term course of antibiotics (>12 weeks)

○ Intranasal corticosteroids

○ Systemic corticosteroids

○ Local decongestants

○ FESS (Functional endoscopic sinus surgery)

○ Other (please give further explanation);……………………………..………………………………………………

…………………………………………………………………………………………………………………………………………………………….

***Clinical case 4***The 51-year-old patients with nasal polyps from casus 1 has underwent endoscopic sinus surgery. However, after 6 weeks, his complaints have returned. On nasal endoscopy, polyps and purulent discharge are visible lateral to the middle turbinate.

**4.1 How would you treat this patient? (multiple answers possible)**

○ No treatment required

○ Nasal saline irrigation

○ Short course of antibiotics (<4 weeks)

○ Long term course of antibiotics (>12 weeks)

○ Intranasal corticosteroids

○ Systemic corticosteroids

○ FESS (Functional endoscopic sinus surgery)

○ Other (please give further explanation);……………………………..………………………………………………

…………………………………………………………………………………………………………………………………………………………….

**Thank you for your cooperation!**
